# Predictive biomarkers of intra-ocular pressure decrease after cataract surgery associated with trabecular washout in patients with pseudo exfoliative glaucoma

**DOI:** 10.1038/s41598-024-53893-5

**Published:** 2024-06-12

**Authors:** Rodolphe Vallée, Enrico Meduri, Jean-Noël Vallée, Athena Lallouette, Zakarya Haffane, Archibald Paillard, Kaweh Mansouri, André Mermoud

**Affiliations:** 1 Diagnostic and Functional Neuroradiology and Brain stimulation Department, 15-20 National Vision Hospital - Paris University Hospital Center, University of PARIS-SACLAY - UVSQ, Paris, France; 2 Mathematics and Applications Laboratory (LMA), CNRS UMR7348, LRCOM i3M-DACTIMMIS, University of Poitiers, Poitiers, France; 3 Glaucoma Research Center, Montchoisi Clinic, Swiss Visio, Lausanne, Switzerland; 4 Ophthalmology Department, Colorado University Medical School, Denver, CO USA

**Keywords:** Goniowash, Irrigation cannula, Pseudo exfoliation, Cataract, Glaucoma, Biomarker, Medical research, Predictive markers

## Abstract

To investigate biomarkers of intra-ocular pressure (IOP) decrease after cataract surgery with trabecular washout in pseudo-exfoliative (PEX) glaucoma. A single-center observational prospective study in PEX glaucoma patients undergoing cataract surgery with trabecular washout (Goniowash) was performed from 2018 to 2021. Age, gender, visual acuity, IOP, endothelial cell count, central corneal thickness, medications, were collected over 16-month follow-up. Multivariable binomial regression models were implemented. 54 eyes (35 subjects) were included. Mean preoperative IOP (IOP_BL_) was 15.9 ± 3.5 mmHg. Postoperative IOP reduction was significant at 1-month and throughout follow-up (p < 0.01, respectively). IOP_BL_ was a predictive biomarker inversely correlated to IOP decrease throughout follow-up (p < 0.001). At 1 and 12 months of follow-up, IOP decrease concerned 31 (57.4%) and 34 (63.0%) eyes with an average IOP decrease of 17.5% (from 17.6 ± 3.1 to 14.3 ± 2.2 mmHg) and 23.0% (from 17.7 ± 2.8 to 13.5 ± 2.6 mmHg), respectively. Performance (AUC) of IOP_BL_ was 0.85 and 0.94 (p < 0.0001, respectively), with IOP_BL_ threshold ≥ 15 mmHg for 82.1% and 96.8% sensitivity, 84.2% and 75.0% specificity, 1.84 and 3.91 IOP decrease odds-ratio, respectively. All PEX glaucoma patients with IOP_BL_ greater than or equal to the average general population IOP were likely to achieve a significant sustainable postoperative IOP decrease.

## Introduction

Pseudo exfoliation (PEX) disease is a common chronic systemic disease among the elderly. It is outlined by the progressive formation and accumulation of abnormal fibrillar, extracellular deposits in diverse organs and tissues, especially the anterior structures of the eye^1^. Ocular involvement consists of chronic accumulation of PEX material deposits on the ciliary body, the zonules, the anterior lens surface, the iris, the pupil edge, the corneal endothelium, in the iridocorneal angle (ICA), on the trabecular meshwork (TM) and in Schlemm’s canal^1^.

The PEX material is in the form of a highly glycosylated, cross-linked, and enzymatically resistant glycoprotein-proteoglycan complex, composed of a protein core surrounded by glycoconjugates^2^. It is multifocally produced by intraocular cells, such as ciliary epithelial cells, pre-equatorial epithelial lens cells, trabecular and corneal endothelial cells, and iris cells^3^. However, the pathophysiological process is still undetermined. In the human eye, IOP is held in a stable balance through different physiological mechanisms between the aqueous humor production and its drainage^1^. Aqueous humor continuously flows into the anterior chamber of the eye and leaves the eye through the trabecular and uveoscleral pathways with an unimpeded outflow rate of 2.4 ± 0.6 µl/min, i.e. 1.0–1.5% of the anterior chamber volume per minute^4^, maintaining a consistent IOP at 15 ± 3 mmHg. This balance is disturbed in patients with PEX syndrome. PEX material deposits blocking the TM and the Schlemm’s canal, slowly impede the physiological aqueous humor outflow and induce an increase in intraocular pressure (IOP), correlated to the quantity of exfoliation deposits^1^. This elevated IOP is correlated to the risk of developing exfoliative glaucoma, the most common identifiable cause of open-angle glaucoma world-wide and irreversible blindness^1^.

Exfoliative glaucoma is a particularly aggressive secondary open-angle glaucoma. It is characterized by greater IOP levels, greater diurnal fluctuations in IOP, greater rate of optic nerve damage, and lower response to treatment, resulting in the accelerated progression of optic neuropathy and perimetric involvement. PEX syndrome is further identified as a risk factor for many intraocular complications^1^. Patients with exfoliative syndrome have a higher and earlier risk of developing nuclear cataracts^1^. Surgical complications of cataract surgery are 5- to 10- fold greater in ocular PEX syndrome than in standard cataract cases^5^. However, several studies have shown that a decrease in IOP and its stabilization over time improved the prognosis of the visual field in patients with exfoliative glaucoma^6^.

Goniowash is a new technique of washout of PEX material combined with cataract surgery, designed as a new surgical approach to lower intraocular pressure in PEX syndrome^7^. It consists of a simple, additional washout procedure of the TM, ICA and the entire anterior chamber of the eye. This procedure, previously described in an article^7^, is simple, short, and respects the microanatomy of the eye as there are no additional incisions, implants, or sutures other than those required for cataract surgery. Recent retrospective studies^7,8^ have shown that cataract surgery associated with trabecular washout using Goniowash technique in patients with PEX glaucoma, decreased significantly and sustainably the IOP and reduced ocular hypotensive medication (OHM) intake.

The interest of the study was to identify biomarkers that predict the decrease in IOP after cataract surgery combined with trabecular washout in a PEX glaucoma population, and to establish the patients' profile likely to achieve the best expected outcome, in terms of lowering postoperative IOP, as predicted by the biomarkers.

## Methods

A single-center observational prospective study from September 2018 to 2021 (recruitment and follow-up), approved by the Swissethics (Cantonal Ethics Commission for Human Research of the Canton of Vaud (CER-VD) in Switzerland #2018-01403), was carried out to identify biomarkers that predict the decrease in IOP after cataract surgery combined with trabecular washout in patients with PEX glaucoma. The study was carried out according to the declaration of Helsinki guidelines.

### Study population

The study population of our study was extracted from a prospective large-scale glaucoma register (NCT number: NCT04381611) which includes any patient suffering or suspected of glaucoma and does not affect their care, treatment choice or follow-up at Montchoisi Clinic, Lausanne, Switzerland.

Routine clinical examination included best-corrected visual acuity (BCVA) recorded using Snellen chart, dilated fundoscopy, central corneal thickness (CCT, µm) measurement by pachymetry, endothelial cell count (EC) using specular microscopy, IOP (mmHg) measurement using Goldmann applanation tonometry, Humphrey visual field test, optical coherence tomography examination, slit-lamp examination and gonioscopy. Demographics such as age, gender (women/men) and eye (OD/OS), as well as number of ocular hypotensive medications (OHM) were recorded.

#### Inclusion criteria

All patients with PEX glaucoma who were scheduled for routine cataract surgery associated with trabecular washout using the Goniowash technique (Fabrinal, Switzerland)^7^; Patients who were able and willing to provide informed written or verbal consent.

#### Exclusion criteria

Patients who were unable to understand the implications of their inclusion in the study or who were unable or unwilling to give informed consent; missing data, loss to follow-up.

Preoperative baseline data were collected, then postoperatively on day D1, week W1, and months M1, 3, 6, 9, 12 and 16 of the follow up.

The primary (IOP decrease from baseline) and secondary (BCVA and OHM changes from baseline) endpoints were analyzed in a full analysis set that met all eligibility criteria.

### Statistical analysis

Continuous variables such as age, IOP, IOP decrease from baseline, EC, CCT, OHM number were expressed as means and standard deviations (± SD). BCVA values were recorded using the Snellen scale, and Snellen values were converted to logMAR units for statistical analyzes. LogMAR values corresponding to count fingers, hand motion, light perception, or no light perception were substituted with 2.10, 2.40, 2.70 and 3.00 logMAR, respectively^9^. Categorical variables such as gender, eye, IOP decrease from baseline (yes/no) were expressed as frequencies and percentages (%). Calculation using the Z statistic for sample size for paired tests required a sample size of 44 subjects to achieve a power of 90% (β = 10%), a level of two-sided significance α of 5%, for detecting an effect size of 0.5 (maximum variability) between pairs. After adding 10% of the calculated sample size to compensate any study‐related problems, the final sample size of the study was 49. Data analyses were performed on a full analysis set using multivariate regression imputation of missing data. The IOP, BCVA and OHM variables were analyzed on day D1, week W1, and months M1, 3, 6, 9, 12 and 16 of the follow up, and compared to those of preoperative baseline (_BL_). Wilcoxon’s matched pairs signed ranks test was used for pre- and post-operative comparisons of IOP, BCVA, and OHM at each relevant time of follow-up.

Multivariable binomial logistic regression analyses with mixed stepwise selection, adjusted for central corneal thickness covariate, were carried out using a logit function, $\mathit{Ln} (\frac{p}{1-p})$ = logit (*p*), to identify predictive independent biomarkers of the decrease in IOP from the baseline (yes/no). The logit of the probability (p) of the occurrence of the decrease in IOP (Y) is expressed as a function of an intercept β0, of the biomarkers (Xi) attached to their regression coefficients βi and to a noise term ε. It consists of modeling the probability that a decrease in IOP will occur, as follows: $\mathit{Ln} (\frac{p}{1-p})$ = logit (*p*) = $\beta$_0_ + $\beta$_1_X_1_ + $\beta$_2_X_2_ + … + $\beta$_i_X_i_ + $\varepsilon$; ($\beta$_i_ = log (OR(X_i_))); OR(X_i_): the unit odds ratio of the biomarkers (Xi).

During the forward step of mixed variable selection, the maximal p-value was 0.25 for an effect to enter the model. During the backward step the minimal p-value was 0.10 for an effect to be removed from the model.

The adequacy of the model to predict the decrease in IOP from baseline was assessed by the calculation of *R-square* metric (R^2^), which represents the percentage of variance of IOP decrease that can be explained by the biomarkers (Xi) included in the model. Prediction performances of biomarkers were quantified by the area (AUC) under the ROC (receiver operating characteristic) curve. Decision cut-offs for prediction were calculated according to the Youden maximal index, J = maxc [Se(c) + Sp(c)–1], with c: all possible biomarker cut-off values; Sp: specificity; and Se: sensitivity.

For given biomarker values, odds ratios ± 95% CI (confidence interval) of IOP decrease were:

${\text{Odds }}\left( {\text{IOP decrease}} \right) \, = {\text{ e}}^{{({\text{b}}0 \, + {\text{ b1X1 }} + {\text{ b2X2 }} + \, \ldots \, + {\text{ biXi}})}} = {\text{ e}}^{{{\text{b}}0}} {\text{e}}^{{{\text{b1X1}}}} {\text{e}}^{{{\text{b2X2}}}} \ldots {\text{ e}}^{{{\text{biXi}}}} ,$ with i: number of biomarkers; Xi: IOP-lowering biomarkers, as model effects; bi: parameters estimated using maximum likelihood estimation.

For the biomarker Xi, the covariables being constant, the unit odds ratio was:

$$ {\text{OR}}\left( {{\text{X}}_{{\text{i}}} } \right) \, = {\text{ e}}^{{{\text{b}}0}} {\text{e}}^{{{\text{b1X1}}}} {\text{e}}^{{{\text{b2X2}}}} \ldots {\text{ e}}^{{{\text{bi}}({\text{Xi }} + {1})}} )/{\text{ e}}^{{{\text{b}}0}} {\text{e}}^{{{\text{b1X1}}}} {\text{e}}^{{{\text{b2X2}}}} \ldots {\text{ e}}^{{{\text{biXi}}}} = {\text{ e}}^{{{\text{b}}0}} {\text{e}}^{{{\text{b1X1}}}} {\text{e}}^{{{\text{b2X2}}}} \ldots {\text{ e}}^{{{\text{biXi}}}} {\text{e}}^{{{\text{bi}}}} /{\text{ e}}^{{{\text{b}}0}} {\text{e}}^{{{\text{b1X1}}}} {\text{e}}^{{{\text{b2X2}}}} \ldots {\text{ e}}^{{{\text{biXi}}}} = {\text{ e}}^{{{\text{bi}}}} . $$

Lack-of-fit tests for regression models were carried out to assess whether the variables selected in the models provided sufficient information to predict IOP decrease. Likelihood Ratio Chi-Squared Tests were carried out to assess the level of significance of the effect of the whole model to predict IOP decrease. Effect Likelihood Ratio Tests were carried out to assess each effect's significant contribution to the model fit to predict IOP decrease. Data analyses were performed on a full analysis set after multivariate regression imputation of missing data. (SAS 9.1, SAS Institute Inc., Cary, NC).

### Ethical approval

The study involving human participants was approved by the Swissethics (Cantonal Ethics Commission for Human Research of the Canton of Vaud (CER-VD) in Switzerland #2018-01403). The study was carried out according to the declaration of Helsinki guidelines.

## Results

54 eyes of 35 PEX glaucoma patients who underwent routine cataract surgery were consecutively included in the full analysis set based on the inclusion and exclusion criteria. All these patients underwent cataract surgery associated with trabecular washout, and received an anhydrase inhibitor (acetazolamide 250 mg, single or double tablet) approximately 3 and 6 h after surgery to prevent postoperative IOP spikes. Washout was performed using the Goniowash technique in all cases. 5 eyes of 5 PEX glaucoma patients had some missing data, and no patients were lost to follow-up. The demographic synopsis of the study subjects (male: n = 17; female: n = 18; overall mean age: 76.8 ± 6.9 years old) is summarized in Table 1.

**Table 1:** (A) Demographic synopsis of the study subjects.

Number of eyes (*n*)	Number of subjects	Gender			Age (years)				
					Average		Min	Max	Median
(A) Demographic synopsis									
54	35	Male n = 17 (48.6%)			76.8 ± 6.9		62	88	70
		Female n = 18 (51.4%)							

*n* = 54	Baseline	D1	W1	M1	M3	M6	M9	M12	M16
(B) Mean value ± standard deviation									
IOP (mmHg)	15.9 ± 3.5	16.8 ± 4.7	16.5 ± 3.8	14.8 ± 2.6	13.9 ± 2.8	14.7 ± 2.7	14.7 ± 3.3	14.2 ± 2.7	13.6 ± 2.1
* P* value*		0.6576	0.4058	0.0092	< .0001	0.0350	0.0490	0.0042	< .0001
BCVA logMAR	0.308 ± 0.270	0.310 ± 0.238	0.160 ± 0.204	0.108 ± 0.161	0.107 ± 0.161	0.132 ± 0.149	0.142 ± 0.192	0.145 ± 0.192	0.135 ± 0.205
* P* value*		0.8140	< 0.0001	< 0.0001	< 0.0001	< 0.0001	< 0.0001	< 0.0001	< 0.0001
OHMs	1.1 ± 1.0	0.2 ± 0.6	0.3 ± 0.6	0.4 ± 0.6	0.6 ± 0.7	0.4 ± 0.6	0.9 ± 0.9	0.8 ± 0.8	0.8 ± 0.9
* P* value*		< .0001	< .0001	< .0001	0.0001	< .0001	0.0822	0.0586	0.1487

	*n (54)*	D1	W1	M1	M3	M6	M9	M12	M16
(C) Decrease in IOP according to the decision threshold of 15 mmHg of OP_BL_									
IOP_BL_ < 15 (mmHg)	17 (31.48%)	5.0 ± 11.6	3.6 ± 3.2	1.1 ± 2.8	0.2 ± 2.5	1.5 ± 1.6	2.1 ± 2.0	1.8 ± 3.5	0.9 ± 3.0
IOP_BL_ $\ge$ 15(mmHg)	37 (68.52%)	− 1.0 ± 7.3	− 0.7 ± 4.5	− 2.2 ± 3.2	− 3.0 ± 3.4	− 2.4 ± 3.3	− 2.7 ± 4.5	− 3.2 ± 3.4	− 3.8 ± 2.6
* P* value**	–	0.0187	0.0004	0.0003	0.0004	< .0001	< .0001	< .0001	< .0001
(D) Visual acuity (BCVA) according to the decision threshold of 15 mmHg of OP_BL_									
IOP_BL_ < 15 (mmHg)	17 (31.48%)	0.5 ± 0.2	0.6 ± 0.2	0.7 ± 0.2	0.7 ± 0.2	0.6 ± 0.2	0.6 ± 0.3	0.7 ± 0.3	0.7 ± 0.3
IOP_BL_ $\ge$ 15 (mmHg)	37 (68.52%)	0.6 ± 0.3	0.8 ± 0.2	0.9 ± 0.2	0.9 ± 0.2	0.8 ± 0.2	0.8 ± 0.2	0.8 ± 0.3	0.8 ± 0.3
* P* value**	–	0.2438	0.0056	0.0813	0.0391	0.0022	0.0076	0.1088	0.0309

(B) Mean value ± standard deviation of intraocular pressure (IOP), best corrected visual acuity (BCVA logMAR) and Medication (medication number) throughout follow-up. (C,D) Decrease in intraocular pressure (IOP) and improvement of visual acuity (BCVA) according to the decision threshold of 15 mmHg of the predictive biomarker of IOP decrease (IOP_BL_, preoperative IOP) for each relevant time of follow-up after cataract surgery associated with trabecular washout using the Goniowash technique.

*Wilcoxon’s two-tailed signed rank test (matched pairs from baseline).

**Wilcoxon rank sum test (Mann Whitney test).

### Intraocular pressure

Mean preoperative IOP (IOP_BL_) was 15.9 ± 3.5 mmHg. No significant change in IOP was observed at 1 day and 1 week postoperatively. Early transient IOP spikes of 30 mmHg or greater within the first 24 h following cataract surgery was observed in 4 out of 54 eyes (7.4%).

**Figure 1:**
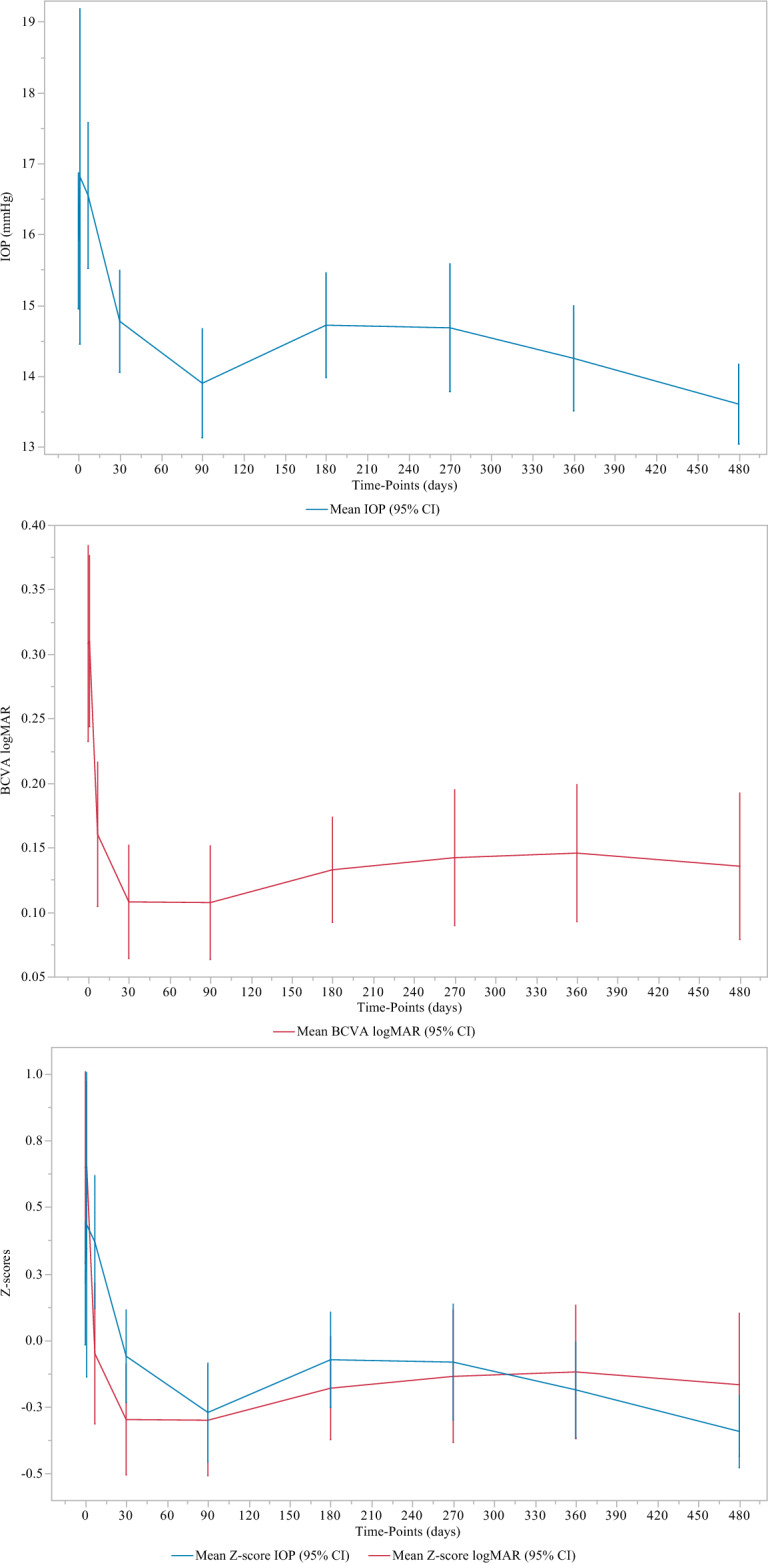
Time effect of cataract surgery associated with trabecular washout using the Goniowash technique on intraocular pressure (IOP mmHg) and best corrected visual acuity (BCVA logMAR) for each relevant time of follow-up.

Postoperative IOP reduction was significant at 1-month and throughout follow-up (p < 0.01, at all visits). Table 1, Fig. 1. At 1, 12 and 16 months of follow-up, the decrease in IOP concerned 31 (57.4%), 34 (63.0%) and 40 (74.1%) eyes with an average decrease in IOP of 17.5% (from 17.6 ± 3. 1 to 14.3 ± 2.2 mmHg), 23.0% (from 17.7 ± 2.8 to 13.5 ± 2.6 mmHg), and 22.1% (from 17.3 ± 2.8 to 13.4 ± 2.1 mmHg), respectively. Table 2.

**Table 2:** Predictive biomarkers and their predictive performance (AUC) obtained from ROCs to predict the decrease in IOP (yes vs no) from baseline for each relevant time of follow-up after cataract surgery associated with trabecular washout using the Goniowash technique.

(n = 54)	D1		W1		M1		M3		M6		M9		M12		M16	
Decrease in IOP	Yes	${\text{No}}$	Yes	${\text{No}}$	Yes	${\text{No}}$	Yes	${\text{No}}$	Yes	${\text{No}}$	Yes	${\text{No}}$	Yes	${\text{No}}$	Yes	${\text{No}}$
IOP_BL_ predictive biomarker of the decrease in IOP (yes vs ${\text{no}}$)																
n (%)	30 (55.6)	24 (44.4)	23 (42.6)	31 (57.4)	31 (57.4)	23 (42.6)	39 (72.2)	15 (27.8)	33 (61.0)	21 (38.9)	27 (50.0)	27 (50.0)	34 (63.0)	20 (37.0)	40 (74.1)	14 (25.1)
IOP_BL_ (mmHg)	17.1 ± 3.4	14.5 ± 3.2	18.2 ± 3.3	14.2 ± 2.6	17.6 ± 3.1	13.7 ± 2.8	16.9 ± 3.2	13.5 ± 3.1	17.5 ± 3.0	13.4 ± 2.6	17.9 ± 2.9	13.9 ± 2.8	17.7 ± 2.8	12.8 ± 2.2	17.3 ± 2.8	11.9 ± 1.7
P value*	0.0031		0.0014		0.0013		0.0355		0.0028		0.0005		< .0001		< .0001	
IOP (mmHg)																
IOP (mmHg)	12.6 ± 3.0	22.1 ± 10.5	14.8 ± 3.0	17.9 ± 3.8	14.3 ± 2.2	15.3 ± 3.1	13.3 ± 1.8	15.5 ± 4.3	14.3 ± 2.5	15.4 ± 2.9	13.2 ± 2.1	16.1 ± 3.6	13.5 ± 2.6	15.5 ± 2.6	13.4 ± 2.1	14.2 ± 1.9
Decrease in IOP																
(mmHg)	− 4.5 ± 3.5	+ 7.6 ± 9.8	− 3.4 ± 2.3	+ 3.6 ± 3.3	− 3.3 ± 2.5	+ 1.7 ± 2.3	− 3.6 ± 2.5	+ 2.0 ± 2.2	− 3.2 ± 2.6	+ 2.0 ± 1.4	− 4.7 ± 3.2	+ 2.2 ± 2.3	− 4.2 ± 2.7	+ 2.6 ± 2.0	− 3.9 ± 2.2	+ 2.4 ± 1.3
(%)	− 24.9	52.9	− 18.3	27.6	− 17.5	14.18	− 19.6	14.6	− 17.3	15.6	− 24.9	17.0	− 23.0	21.8	− 22.1	20.8
Prediction performance (AUC) of IOP_BL_ biomarker																
AUC	0.837		0.808		0.851		0.811		0.810		0.835		0.940		0.976	
P value**	0.0003		0.0006		< .0001		0.0025		0.0006		< .0001		< .0001		< .0001	
Decision threshold	15 (mmHg)		17 (mmHg)		15 (mmHg)		15 (mmHg)		15 (mmHg)		15 (mmHg)		15 (mmHg)		15 (mmHg)	
Se	82.14		76.19		82.14		71.43		87.10		80.00		96.77		94.59	
Sp	89.47		80.77		84.21		83.33		68.75		77.27		75.00		100.00	
Odds ratios (OD)	1.53		1.66		1.84		1.61		1.71		1.77		3.91		6.12	
95% IC (lower)	1.16		1.24		1.33		1.19		1.26		1.30		1.99		2.32	
95% IC (upper)	2.15		2.43		2.84		2.40		2.56		2.67		12.43		36.21	

*Multivariate regression analyses with mixed stepwise selection, adjusted for central corneal thickness covariate.

**Logistic regression models.

### Best-corrected visual acuity

Improvement in BCVA was significant at 1 week postoperatively and throughout follow-up, (p < 0.0001, at all visits). Table 1, Fig. 1. Average BCVA improved from 0.5 ± 0.3 to 0.8 ± 0.3 at 1-week follow-up and remained stable throughout follow-up.

### Mean number of OHMs

The mean number of OHMs decreased throughout the follow-up, significantly at 1-day and up to 6-months of follow-up (p =  < 0.0001, respectively) then with a trend towards significance at M9 and M12 of the follow up, (p = 0.0822 and 0.0586, respectively) Table 1.

### Predictive biomarkers of decrease in IOP

To predict the decrease in IOP from the baseline during the follow-up, multivariable binomial logistic regression models, adjusted for the CCT covariate, tested the effects of the variables age, sex, eye, EC, IOP_BL_, and variations in number of OHM at each relevant time of follow-up. IOP_BL_ was a predictive biomarker inversely correlated to the decrease in IOP on day D1, week W1, and month M1, 3, 6, 9, 12 and 16 of the follow up, (p < 0.05, respectively). Table 2. Age, sex, eye, endothelial cell count, changes in OHM from baseline were not independent predictive factors of the IOP decrease during follow-up.

AUCs obtained from ROCs, optimal decision thresholds and unit odds ratios for the preoperative classifier IOP_BL_ to predict the decrease in IOP (yes vs no) for each relevant time of follow-up are summarized in Table 2.

**Figure 2:**
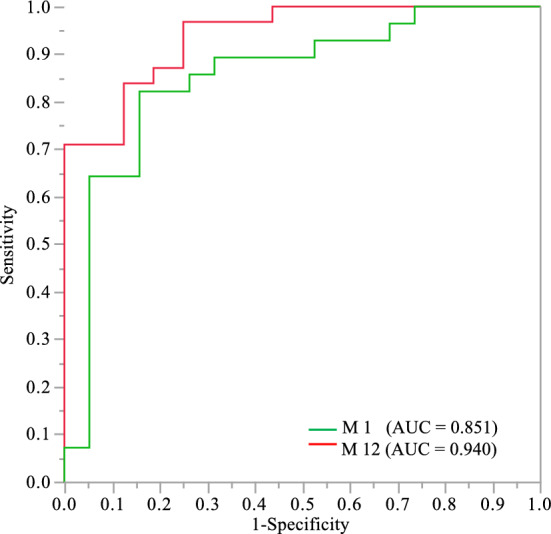
Prediction performance (AUC) of the IOP_BL_ biomarker (preoperatory intraocular pressure) for predicting the decrease in IOP (< 0 vs ≥ 0) from baseline at M_1_ and M_12_ months follow-up after cataract surgery associated with trabecular washout using the Goniowash technique.

At 1- and 12-months follow-up, the predictive performance (AUC) of the IOP_BL_ classifier was 0.85 and 0.94 (p < 0.0001, respectively), with an IOP_BL_ cut-off value ≥ 15 mmHg, for 82.1% and 96.8% sensitivity, 84.2% and 75.0% specificity, and an odds ratio of IOP decrease of 1.84 and 3.91, respectively. Table 2, Fig. 2.

**Figure 3:**
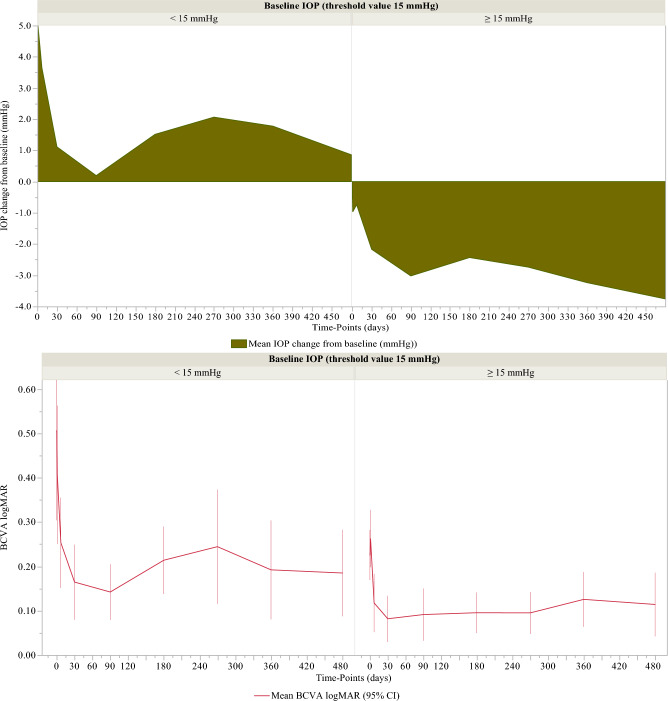
Decrease in intraocular pressure (IOP) and best corrected improvement of visual acuity (BCVA logMAR) according to the decision cut-off value of 15 mmHg of the predictive biomarker of IOP decrease (IOP_BL_, preoperative IOP) for each relevant time of follow up after cataract surgery associated with trabecular washout using the Goniowash technique.

According to the cut-off value of 15 mmHg of the predictive biomarker IOP_BL_, the decrease in IOP and improvement of BCVA were significant at 1-day and 1-week follow-up, (p = 0.0187 and 0.0056) respectively, then throughout follow-up (p < 0.001 and < 0.05, respectively) Table 1, Fig. 3.

At 1- and 12-months follow-up, conclusions for AUC of the IOP_BL_ and IOP_BL_ cut-off value were relatively similar in both the full analysis set with missing data imputation and the set of complete-case analysis in which AUC was 0.85 and 0.94 (p < 0.0001, respectively), with an IOP_BL_ cut-off value ≥ 15 mmHg, for 82.1% and 93.3% sensitivity, 83.3% and 78.6% specificity, and an odds ratio of IOP decrease of 1.80 and 3.72, respectively.

## Discussion

The identification of predictive biomarkers is helpful to maximize patients’ chances of reaching the expected outcome of the treatment predicted by the biomarkers. Predictive models of multivariable binomial stepwise logistic regression were implemented to establish the patients’ profile with PEX glaucoma likely to achieve the best expected outcome, in terms of decrease in IOP, after cataract surgery. Logistic regression consists in modeling the probabilities of having a decrease in IOP using logit functions. The stepwise selection process was used to select a subset of effects for a regression model, knowing that there is little insight in the literature to guide the selection of terms of a model for the prediction of the decrease in IOP. This process allows to interactively explore which predictors seem to provide a good fit for the model. The control of effects entering and leaving the models was ensured by a mixed direction of stepwise selection that alternates the forward and backward steps. The stepwise model incorporates the most significant term relative to the maximum p-value threshold and removes the least significant term relative to the minimum p-value threshold. The term removing process carries on until the next terms are significant to change to the forward direction. The multivariate regression models were adjusted for the central corneal thickness covariate, a confounding variable affecting the accuracy of applanation-measured IOP^10^. The modeling results showed that preoperative IOP (IOP_BL_) reliably and sustainably predicted the decrease in postoperative IOP with a high predictive performance, sensitivity and specificity.

Model convergence was reached. The whole model effect in predicting the decrease in IOP was significant *(*p < *0.0001)* and effective* (R-square* = *0.612)*, which demonstrated that the model significantly explained the variance of the IOP decrease, and reflected the variables of age, sex, eye, endothelial cell count, baseline IOP and medications. The absence of statistical significance (*p* = *0.617*) of the Chi-Squared Test of Lack-of-fit for the whole regression model signified that there was no lack-of-fit of the model. Therefore, the variables selected in the models provided sufficient information to predict IOP decrease. Introducing supplementary terms into the model, such as using polynomials or cross terms, would not significantly improve the model fit. This was consistent with the significant contribution of the effect of preoperative IOP (IOP_BL_) to the model fit (p = 0.0016). This demonstrated that the parameter IOP_BL_ contributed significantly and usefully to improve the model’s performance of predicting the decrease in IOP.

The regressor for IOP_BL_ was positive which indicated that the decrease in IOP increased when the value of IOP_BL_ increased by 1 unit. At 1- and 12-months follow-up, the values of 1.84 and 3.91 of unit odds ratios of IOP decrease from baseline indicated that the probabilities of IOP reduction were 1.84 and 3.91 times greater, respectively, for an increment of + 1 mm in IOP_BL_.

ROC curve area (AUC), or accuracy index, is a metric of performance to quantify the IOP_BL_ classifier’s accuracy of discrimination between two patients’ postoperative states, namely "IOP decrease" and "no IOP decrease ". This represented the probability that a subject with a decrease in IOP, randomly selected, is rated as more likely to have a decrease in IOP than a randomly chosen subject with no decrease in IOP. This was based on non-parametric Wilcoxon-Mann–Whitney U statistics performed in the calculation of the AUC.

This prospective study showed that cataract surgery associated with trabecular washout provided sustainable IOP decrease with a postoperative IOP of less than 15 mmHg over a 16-month follow-up period, indicating a postoperative recovery of normal physiological flow of the aqueous humor throughout the follow-up in these patients with PEX glaucoma. Goniowah technique using the Tran Canula®^7,8^ is designed to remove macroscopic and microscopic pseudo exfoliative deposits and pigmented cells located in the entire anterior chamber of the eye, and to restore the physiological pathway of aqueous humor through the trabecular meshwork and the iridocorneal angle. The design of the Tran Canula® is characterized by two distinct water jets separated from each other by an angle of 30°. Thanks to the technological specificity of the cannula and to micro-hydrodynamic laws, these two independent fluid streams merge into one more powerful since they encounter an obstacle in front of them. So, the water flow goes straight ahead, really targets the trabecular meshwork and the iridocorneal angle, and focuses precisely on the area to be cleaned. Our results were consistent with previous studies^7,8,11–21^, and more especially with a recent review^22^ of six randomized controlled trials, including a total of 639 eyes with gonioscopically confirmed primary and secondary open-angle glaucoma^23,24^, that highlighted a statistical difference in IOP and need for glaucoma medication between before and after cataract surgery. The mechanisms responsible for IOP reduction after surgery remain to be elucidated. They can be molecular, physiological and biomechanical^25,26^. Among the biomechanical mechanisms, fluidics might play a role. Per-operatively washing and rinsing with high flow rates the trabecular meshwork, the irido-corneal angle and the entire anterior segment, such as during the Goniowash procedure, facilitates the elimination of most macro- and microscopic pseudo exfoliation deposits and/or pigmented cells^25,27–29^. In the setting of PEX glaucoma, previous studies reported greater IOP decrease in patients in which greater irrigation volumes were used intraoperatively^15,30,31^. The magnitude of IOP reduction correlated with the increased aspiration time and the irrigation fluid volume used during the intervention^30,32,33^.

Additionally, preoperative IOP (IOP_BL_) was a predictive biomarker of postoperative IOP decrease with a high prediction performance, sensitivity and specificity in PEX glaucoma patients with preoperative IOP ≥ 15 mmHg. This threshold of 15 mmHg represents the mean IOP of the normal physiological flow of the aqueous humor. Thus, over a 16-month follow-up, all PEX glaucoma patients with a preoperative IOP greater than or equal to the average general population IOP were likely to achieve, according to a prediction statistical relationship, a significant sustainable decrease in postoperative IOP; the higher the preoperative IOP, the greater the decrease in postoperative IOP. Age and gender were not predictive factors associated with IOP decrease. The results of our study were consistent with the few studies that investigated the predictors of lowering IOP after cataract surgery^34–38^ and more specifically with the rare predictive studies in the setting of the non-glaucoma PEX syndrome, in 33 patients^32^, and PEX glaucoma, in 29 patients^30^. However, these studies did not provide decision threshold of the intraocular pressure, with a prediction performance, sensitivity, and specificity, to establish the patients' profile likely to achieve the best expected outcome, in terms of decrease in intraocular pressure, after cataract surgery, for a personalized decision-making in care of patients, especially of patients with pseudo exfoliation glaucoma. The threshold of about 15 mmHg for IOP-reducing effect has been shown for both topical medication with prostaglandin analogues (latanoprost)^39^ in newly detected open-angle glaucoma patients and laser trabeculoplasty^40^ in newly diagnosed multi-treated glaucoma patients. Both treatments affect the outflow of aqueous humor. The threshold of about 15 mmHg seems to corroborate the restitution of the physiological flow of aqueous humor.

In our study, the incidence of early transient IOP spike of 30 mmHg or greater within the first 24 h following cataract surgery was observed in 7.4% of patients. Previous studies showed that these IOP spikes were more common in PEX eyes than in non-PEX eyes^14,15,41^. This incidence has been reported between 11 and 35% in ocular PEX patients and between 3 and 27% in primary open-angle glaucoma patients^15,16^. IOP spike may have a harmful impact on the remaining nerve fibers, leading to further loss of visual field^42^.

One limitation of the study was the population size (54 eyes), although similar to previous studies^30,32^ which included 33 and 29 eyes, respectively. Another limitation was the absence of discontinuation of hypotensive drugs before and after surgery, precluding the assessment of the real baseline IOP and underestimating IOP reduction following surgery^43^. Anterior chamber biometric factors were not included in the models. However, they did not demonstrate significant associations with changes in postoperative IOP in previous studies^32^, which could bias the models' adjustment. The strength of the study was its prospective design; the assessment of the optimal decision threshold of the predictive biomarker, with its prediction performance, sensitivity, specificity and odds ratio, for the identification of a patients' profile likely to achieve the expected outcome, in terms of decrease in postoperative IOP, in a population with PEX glaucoma.

## Conclusion

All PEX glaucoma patients with a preoperative IOP greater than or equal to the average general population IOP (≥ 15 mmHg) were likely to achieve a significant sustainable decrease in IOP after cataract surgery associated with trabecular washout using the Goniowash technique. This biomarker may be useful for a personalized approach in care of those patients with particularly aggressive glaucoma, and eventually delaying the progression of ocular PEX disease.

## Data Availability

The datasets used and/or analyzed during the current study available from the corresponding author on reasonable request.

## Abbreviations

IOP: Intraocular pressure
BL: Preoperative baseline
PEX: Pseudo exfoliation
ICA: Iridocorneal angle
TM: Trabecular meshwork
OHM: Ocular hypotensive medication
VCVA: Best corrected visual acuity
EC: Endothelial cell count
CCT: Central corneal thickness
SD: Standard deviation
ROC: Receiver operating characteristic
AUC: Area under the ROC curve
CI: Confidence interval
